# Predicting risk of obesity in overweight adults using interpretable machine learning algorithms

**DOI:** 10.3389/fendo.2023.1292167

**Published:** 2023-11-17

**Authors:** Wei Lin, Songchang Shi, Huibin Huang, Junping Wen, Gang Chen

**Affiliations:** ^1^ Department of Endocrinology, Shengli Clinical Medical College of Fujian Medical University, Fujian Provincial Hospital, Fuzhou, China; ^2^ Department of Critical Care Medicine, Shengli Clinical Medical College of Fujian Medical University, Fujian Provincial Hospital South Branch, Fujian Provincial Hospital Jinshan Branch, Fujian Provincial Hospital, Fuzhou, China

**Keywords:** obesity risk prediction, machine learning algorithm, overweight, Shapley additive explanation (SHAP) values, CatBoost algorithm

## Abstract

**Objective:**

To screen for predictive obesity factors in overweight populations using an optimal and interpretable machine learning algorithm.

**Methods:**

This cross-sectional study was conducted between June 2011 and January 2012. The participants were randomly selected using a simple random sampling technique. Seven commonly used machine learning methods were employed to construct obesity risk prediction models. A total of 5,236 Chinese participants from Ningde City, Fujian Province, Southeast China, participated in this study. The best model was selected through appropriate verification and validation and suitably explained. Subsequently, a minimal set of significant predictors was identified. The Shapley additive explanation force plot was used to illustrate the model at the individual level.

**Results:**

Machine learning models for predicting obesity have demonstrated strong performance, with CatBoost emerging as the most effective in both model validity and net clinical benefit. Specifically, the CatBoost algorithm yielded the highest scores, registering 0.91 in the training set and an impressive 0.83 in the test set. This was further corroborated by the area under the curve (AUC) metrics, where CatBoost achieved 0.95 for the training set and 0.87 for the test set. In a rigorous five-fold cross-validation, the AUC for the CatBoost model ranged between 0.84 and 0.91, with an average AUC of ROC at 0.87 ± 0.022. Key predictors identified within these models included waist circumference, hip circumference, female gender, and systolic blood pressure.

**Conclusion:**

CatBoost may be the best machine learning method for prediction. Combining Shapley’s additive explanation and machine learning methods can be effective in identifying disease risk factors for prevention and control.

## Introduction

Obesity and being overweight have become global public health concerns. In 2016, more than 1.9 billion adults were overweight and there were approximately 650 million people with obesity (1). The prevalence of overweight/obesity shows an increasing trend (2). Notably, this increase is particularly dramatic in developing countries (3). Epidemiologic studies have identified strong adverse associations between obesity and an expanding set of chronic diseases (4), such as type 2 diabetes (5), cardiovascular disease (6), musculoskeletal disorders (7), and certain cancers (6). Owing to the presence of such pandemic diseases, the prevention and control of obesity is difficult and complicated.

Being overweight is a precursor to obesity. According to the staging system of the American Association of Clinical Endocrinologists’ 2014 advanced framework for a new diagnosis of obesity as a chronic disease (8), a four-stage approach is recommended. Overweight with or without related sub-clinical conditions was classified as stage one or stage two (8). Early intervention for individuals with overweight is more efficacious and feasible for preventing obesity. Therefore, being overweight, not obese, is an actionable and measurable target in national health policies. As the global health community works to develop treatments and prevention policies to address obesity, timely information about individuals with overweight who are inclined to develop morbid obesity is needed (3). The easy identification of overweight individuals in high-risk classes in the absence of any disease could promote early identification and thus reduce the prevalence of those with metabolic syndrome who are likely to become obese.

Machine learning has received significant attention owing to its excellent ability to perform reliable predictive analysis (9–11). Compared with traditional methods (12), recent studies have indicated the applications of machine learning in the analysis of high-dimensional datasets and the complex relationships between many multiple variables (13). Hitherto, most previous machine learning models have focused on the prediction of childhood obesity (14). Studies on the application of machine learning for obesity prediction in the overweight adult population are scarce.

Additionally, machine learning offers great advantages in building predictive models to identify risk factors, but there are still several consensus problems, including cross-validation and overfitting, or poor interpretability of prediction models (15). Thus, in this study, seven commonly used machine learning methods were applied to construct obesity risk prediction models in overweight adult population. The best method was selected by verifying the accuracy and validity of the model. Finally, the model was visualized and explained using Shapley additive explanation (SHAP) values to screen for common but significant obesity predictive factors (16).

## Materials and methods

### Study population

**Figure 1:**
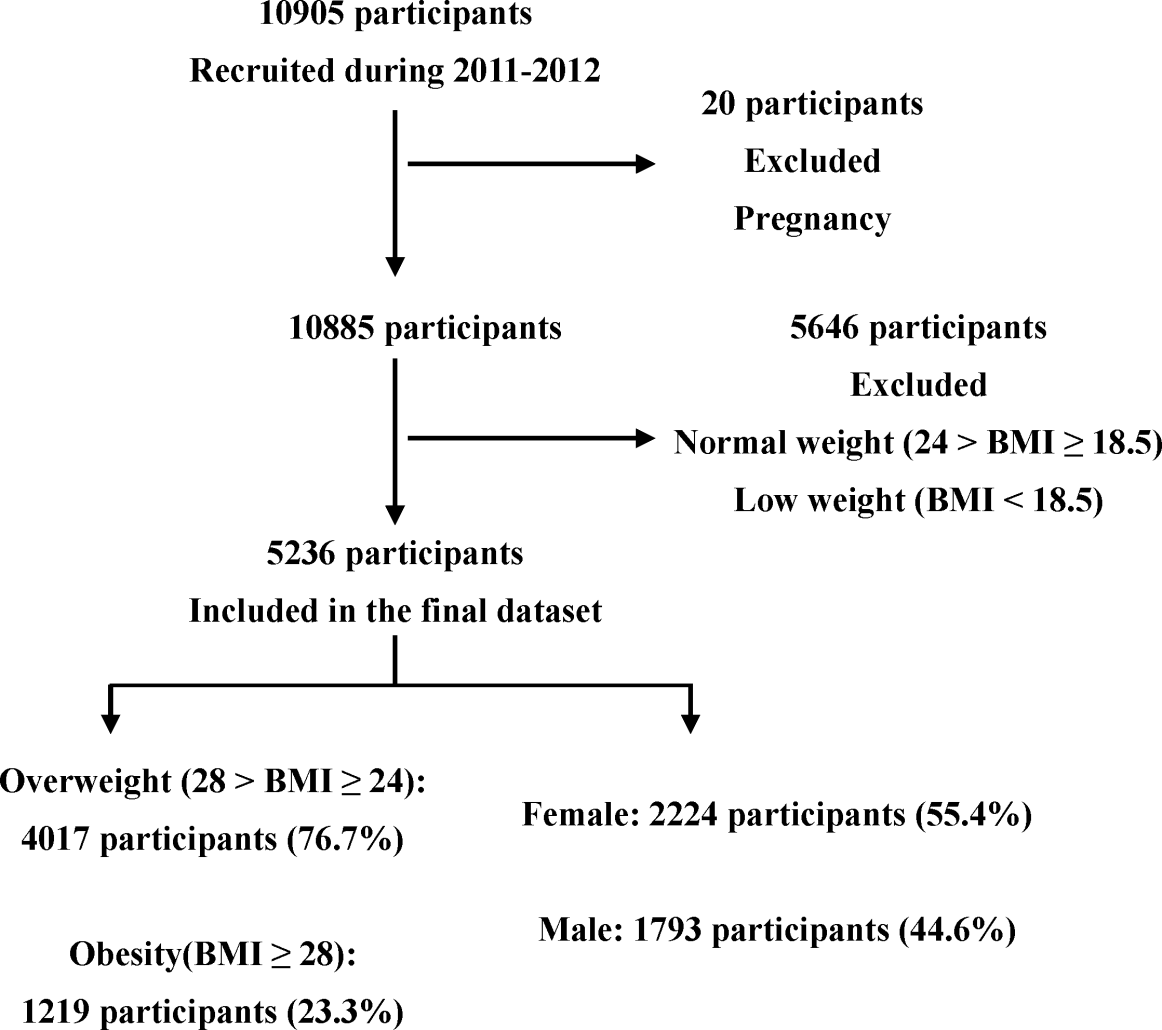
Flowchart of selection for participants.

This cross-sectional study was conducted between June 2011 and January 2012. The participants were randomly selected using a simple random sampling technique. Overall, 10,905 Chinese participants (aged 18–84 years) from Ningde City, Fujian Province, China, participated in this study. Body mass index (BMI) was calculated as the weight in kilograms divided by the height in meters squared. Obesity (12, 17) was defined as BMI ≥ 28; overweight as 28 > BMI ≥ 24; normal weight as 24 > BMI ≥ 18.5; and low weight as BMI < 18.5. (3) The inclusion criteria were individuals who were defined as overweight or obese. The exclusion criteria were individuals who were pregnant, or who had a normal or low body weight. **Figure 1** shows the flowchart of patient selection.

### Data collection

All participants were required to complete a standard questionnaire on age, sex, personal medical history, and habits. Further, the height, waist circumference (WC), hip circumstance (HC), and weight were measured by nurses with ten years of experience, and measured to 0.1 cm, 0.1 cm, 0.1 cm, and 0.1 kg, respectively (12). WC was measured at the middle point of the iliac crest and costal margin. The HC was measured at the largest circumference around the hips.

Heart rate, systolic blood pressure (SBP), and diastolic blood pressure (DBP) were measured three times using a standard electronic sphygmomanometer (OMRON HEM-7111, Kyoto, Japan), and the mean of the three readings was used for analysis. Hypertension was defined as SBP ≥140 mmHg and/or DBP ≥90 mmHg or the use of antihypertensive medications (12).

### Biochemical evaluation

Blood samples were collected following an 8- to 12-h overnight fast and stored at −20°C until analysis. Blood samples were evaluated at the laboratory of the Ningde Municipal Hospital. Blood glucose levels were determined by the glucose oxidase method (Sclavo, Siena, Italy). Fasting insulin was measured using an electrochemiluminescence immunoassay with an Elecsys 1010 immunoassay analyzer (Roche Diagnostics). Type-2 DM was defined (18) as fasting blood glucose (FBG) ≥ 7.0 mmol/L, 2-h postprandial blood glucose (PBG) ≥ 11.1 mmol/L, previous diagnosis of type-2 DM, or use of hypoglycemic medications (18). Insulin resistance from fasting insulin and glucose was calculated using the following formula: Homeostasis Model Assessment of Insulin Resistance (HOMA-IR) = Fasting Insulin (µU/ml) Fasting Glucose (mg/dl)/(22.5 × 18) (12). HbA1c was measured by high-performance liquid chromatography using the VARIANT II Hemoglobin Testing System (Bio-Rad, China) in the National Glycohemoglobin Standardization Program certified central laboratory.

In addition, an automatic colorimetric method (Hitachi, Boehringer Mannheim) was used to measure total triglycerides (TG), total cholesterol (TC), and high-density lipoprotein cholesterol (HDL-C), whereas low-density lipoprotein cholesterol (LDL-C) was detected using the Friedewald formula. Hyperlipidemia was defined as self-reported current treatment with cholesterol-lowering medication or having one or more of the following: TC ≥ 5.17 mmol/l, TG ≥ 1.69 mmol/l, HDL-C ≤ 1.03 mmol/l, or LDL-C ≥ 3.38 mmol/l. Chronic nephrosis was defined as an abnormal glomerular filtration rate (GFR) < 60 ml/min/1.73 m² or previous diagnosis of chronic kidney disease.

### Analysis of missing values

Thirty-two clinical features were included in the dataset, including the outcome variables. The linear correlation of continuous numerical variables in the dataset was performed to avoid the influence of significant correlation characteristics on later model construction. An exploration of the distribution of the population data for the final dataset is provided in **Supplementary Data 1**. As shown in **Supplementary Data 2**, the following two pairings were remarkably correlated: CHOL and LDL (r = 0.86), and HOMA-IR and fasting insulin (FINS) (r = 0.92). Therefore, two variables, CHOL and FINS, were excluded on the basis of clinical experience.

Categorical variables were analyzed using virtual packages. Thirty observation indicators were analyzed for missing values. K-nearest neighbor (KNN) algorithm from Python was used to fill in missing data (n = 5). The distribution of missing values is shown in **Supplementary Data 3**.

### Preprocessing of dataset

Data standardization is a prerequisite for machine learning. StandardScaler, a preprocessing module, was used to standardize the data in this study (19).

SelectFromModel (20) is a transformer that can be employed for feature selection to improve the accuracy scores of estimators or boost their performance on very high-dimensional datasets. In this study, an L1-based feature selection was used (penalty = “L2,” c = 0.05, n=10). As the desired number of features was set to ten, the procedure was recursively repeated on the pruned set until the ten best features were selected.

### Statistical analysis

Continuous numerical variables satisfying a normal distribution are described as the mean ± SD. Those without a normal distribution are represented by the median (lower quartile, upper quartile). Categorical variables are expressed as a sum (percentage).

To select the optimum machine learning algorithmic program to predict obesity, seven machine learning algorithms were employed: logistic regression, KNN, artificial neural network/multiparametric linear programming (ANN/MLP), decision tree, random forest, gradient boosting machine (GBM), and CatBoost. The prediction capacity of the seven-machine learning algorithmic programs were evaluated using the score of the test set, precision, recall, f1 scores, accuracy, confusion matrices, receiver operating characteristic (ROC) curves, cross-validation, and decision curve analysis (DCA) combined with a calibration curve. Precision assesses the number of positive predictions as actual positive observations. Recall accesses the number of actual positive observations that are properly predicted. f1 score is an ‘average’ for both precision and recall. In addition, accuracy assesses how good a machine learning algorithm is. Using the optimal machine learning algorithm, the importance of all risk factors under study was assessed using the scikit-learn feature selection method and the SHAP tool.

The R software (version 3.6.3) was used to preprocess the data. Preparation, construction, evaluation, and visualization of the machine learning data were performed using Python software (version 3.7). The data were organized in the format required for implementing the machine learning algorithm. The KNN, Sklearn (20), and SHAP packages were applied using Python software (version 3.7) to fill in the missing data, build and verify the prediction models, and visualize and explain the models, respectively. The models were constructed using the scikit-learn package (20). An exhaustive grid search was performed to search the hyper-parameter space for the best cross-validation score in the ANN/MLP, decision tree, and random forest models (20).

## Results

### Participant characteristics

The data of 10,905 participants were preprocessed and screened using R software. According to the inclusion and exclusion criteria, the data of 5,236 participants with overweight were obtained as the final dataset. The dataset was divided into training and test sets in a ratio of 2:1 (3,665 and 1,571 for the training and test sets, respectively) (**Supplementary Data 4**). The reason for this was to balance the partition choice for the large dataset size and relatively small amount of obesity.

### Construction and evaluation of models

**Figure 2:**
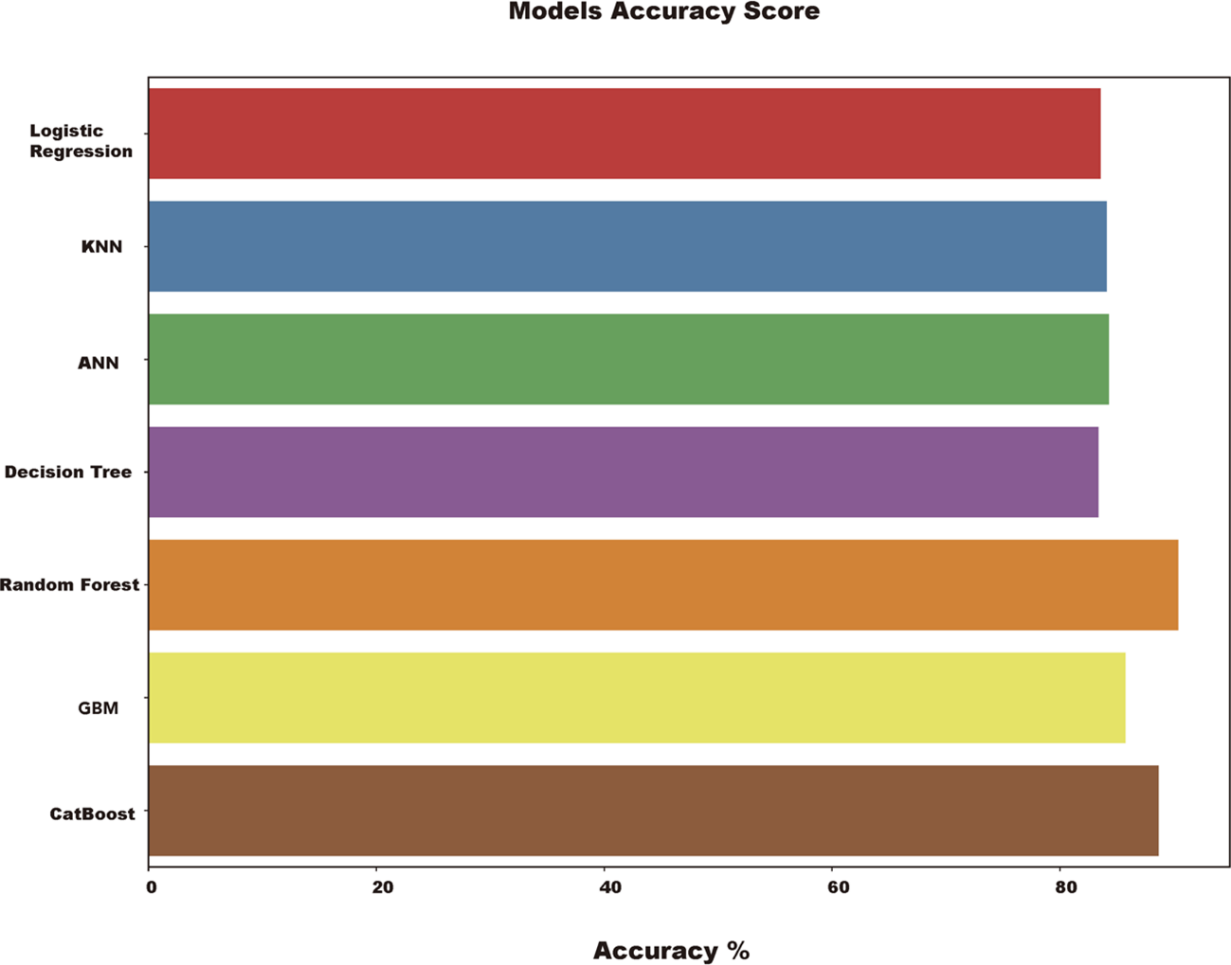
Models Accuracy Score.

Metrics and scoring for quantifying the quality of predictions for seven machine learning algorithms are summarized in **Table 1**. The CatBoost algorithm displayed the best test set and f1-score. As shown in **Figure 2**, by comparing the results of all models, the random forest and CatBoost models exhibited a relatively higher accuracy. Furthermore, to qualify the discriminative capacity of the model, confusion matrices, ROC curves, cross-validation, and DCA combined with a calibration curve were performed.

**Table 1:** Metrics and scoring for quantifying the quality of predictions.

	Scores		Precision		Recall		f1-scores	
	Train set	Test set	Overweight	Obesity	Overweight	Obesity	Overweight	Obesity
Logistic regression	0.85	0.84	0.85	0.76	0.95	0.50	0.90	0.60
KNN	0.87	0.82	0.83	0.73	0.95	0.42	0.89	0.54
ANN/MLP	0.88	0.83	0.83	0.73	0.95	0.42	0.89	0.54
Decision tree	1.0	0.79	0.85	0.76	0.95	0.49	0.90	0.60
Random forest	1.0	0.84	0.86	0.74	0.94	0.53	0.90	0.62
GBM	0.87	0.84	0.85	0.75	0.94	0.52	0.90	0.61
CatBoost	0.91	0.83	0.85	0.75	0.94	0.52	0.90	0.61

KNN, K-nearest neighbor; ANN/MLP, Artificial neural network/Multiparametric linear programming; GBM, Gradient boosting machine.

**Figure 3:**
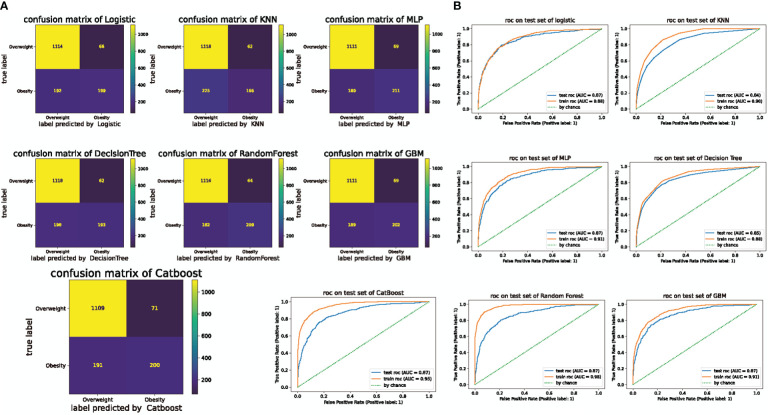
Confusion matrix of machine learning and ROC curves for the test and training sets. **(A)** Confusion matrix for machine learning. The numbers represent the total number of patients. The vertical axis shows the true label and the horizontal axis shows the label predicted by logistic regression. KNN: K-nearest neighbor (KNN), MLP: Artificial neural network/multiparametric linear programming, GBM: gradient boosting machine (GBM). **(B)** ROC curves for the test and training sets in the seven machine learning algorithms.

The confusion matrices for the results of the models trained on the training and test sets are shown in **Figure 3A**. ROC curves are generally used to evaluate prediction models. As shown in **Figure 3B**, the categorization accuracy of the current classifiers was good. Moreover, the area under the curve (AUC) was between 0.84 and 0.87 for the test set.

**Figure 4:**
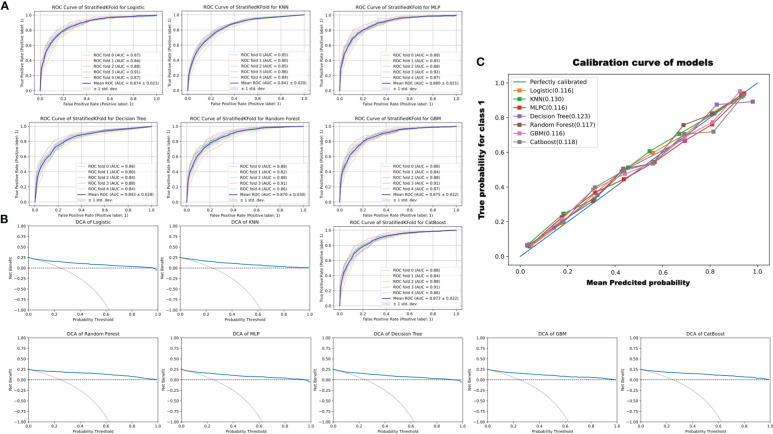
ROC curve of StratifiedKFold for models, decision curve analysis (DCA) to evaluate the clinical benefit of prediction, and the calibration curve of models. **(A)** The ROC curve of 5-StratifiedKFold of different models. **(B)** DCA analysis to evaluate the clinical benefits of prediction. The two dotted lines reflecting the strategies of “assume all patients have the condition” (i.e., treat all) and “assume no patients have the condition” (i.e., treat none) cross at the midpoint of the preference range. In the CatBoost model, a high net benefit is observed across a wide range of threshold probabilities. **(C)** Calibration curves of the models. The reference is a diagonal line, and the calibration curve coincides with the reference when the predicted value is equal to the observed value. The calibration curve was below the reference when the risk was overestimated and above when the risk was underestimated. As can be seen, the predicted values exhibit good performance. The KNN, decision tree, and CatBoost were 0.130, 0.123, and 0.118, respectively. The logistic, MLP, random forest, and GBM were 0.116, 0.116, 0.117, and 0.116, respectively.

K-fold cross-validation is a commonly applicable and powerful method for evaluating discrepancies in model accuracy (11, 20). A five-fold cross-validation was used in this study. In this study, all models performed well, with a mean AUC of ROC > 0.84. Moreover, as shown in **Figure 4A**, the AUC of the CatBoost model was between 0.84 and 0.91 (mean AUC of ROC: 0.87 ± 0.022), and the model performed better than the other models assessed.

Finally, to determine whether the use of a prediction model to inform clinical decision-making would be beneficial, DCA was employed (21). However, some studies have suggested that the AUC is not sensitive enough to predict improvements (22). Therefore, the calibration curve was used for another identification measurement (23) In this study, the net benefit of the decision curve for all the constructed models was higher than that for either “treat all” or “treat none” (**Figure 4B**). The calibration curve is always above the reference dotted line, indicating that the prediction model performs well. KNN and decision tree are 0.130 and 0.123, respectively, and CatBoost is 0.118 (see **Figure 4C**). Thus, the results of DCA combined with the calibration curve suggested that the constructed seven models could be applied to assist clinical decision-making in improving patient outcomes.

All seven machine learning models constructed in this study exhibited good performance. However, upon comparing the accuracy and discriminative capacity of the prediction algorithms, the CatBoost models were found to show the best performance.

### Visualization and explanation of the models

**Figure 5:**
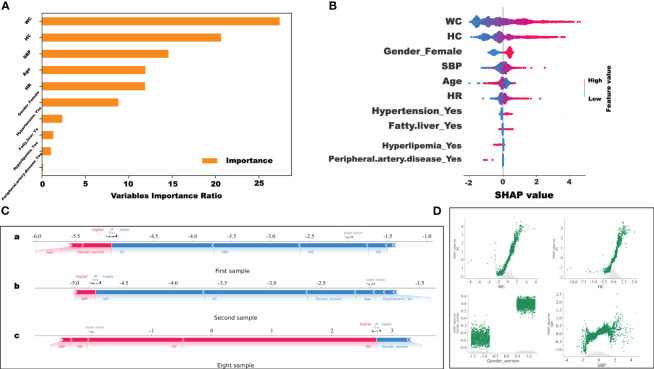
Visualization and explanation of the machine learning models. **(A)** Variable importance ratio output using Scikit-learn. **(B)** For the variable importance output by SHAP, the vertical axis ranks the features according to the sum of the SHAP values (the distribution of the influence of the features on the model output). The base value on the horizontal axis represents the average population SHAP value. Peripheral.artery.disease_Yes: Positive patient history of peripheral artery disease. **(C)** SHAP force plot. The horizontal axis is the SHAP value. Red indicates that the feature has a positive impact on the prediction (arrow to the right, SHAP value increases); blue indicates that the feature has a negative impact on the prediction (arrow to the left, SHAP value decreases). On the number axis, the base value is marked above the horizontal axis, which is the average f(x) value of all samples, and f(x) is marked above the horizontal axis, which is the average SHAP value after the samples are aggregated, that is, the model predicts the mean value. Below the horizontal axis are the features that influence the outcomes of this model. **(D)** SHAP values of WC, HC, female sex, and SBP features, respectively.

The CatBoost prediction models performed best in terms of model validity and clinical net benefit. Therefore, using CatBoost, the importance of all features under study was assessed. The importance ratio of the feature variable, that is, the weight value, was estimated. The higher the value, the higher the weight. In **Figure 5A**, the top three heavily weighted variables are WC, HC, and SBP.

However, the display of such characteristic variables cannot precisely indicate the positive and negative correlations between the features in the model. Thus, the SHAP value was used to visualize the effects of the features in the model. **Figure 5D** shows the SHAP values of the WC, HC, female sex, and SBP features. As shown in **Figure 5B**, the results showed that the higher the WC, the higher the SHAP values. This implies that the WC value has a positive impact on predictive power (**Figure 5B**). The results were like those for HC and SBP. Interestingly, it was found that the higher the HC, the higher the SHAP value. This indicates that WC, HC, and SBP had a positive impact on the prediction model. Moreover, female sex was a positive predictor of obesity in overweight individuals in this study.

More importantly, in this study, the SHAP force plot was used to illustrate the model at the individual level. Through SHAP analysis of individual samples, we screened the high-risk samples and could identify the high-risk patients. As shown in **Figure 5C**, the horizontal axis represents the SHAP value. Red indicates that the feature has a positive impact on the prediction (arrow to the right, SHAP value increases); blue indicates that the feature negatively impacts the prediction (arrow to the left, SHAP value decreases). The base value is marked above the horizontal axis as the average value for all samples, and f(x), also marked above the number axis, represents the average SHAP value after the aggregation of the sample. That is, the model predicts the mean value. As shown in **Figure 5C**, compared to the first (SHAP= - 5.04) (**Figure 5C**-a) and the second (SHAP= - 4.79) (**Figure 5C**-b) samples, the eighth sample (SHAP=2.74) (**Figure 5C**-c) belonged to the high-risk group with a greater risk of developing morbid obesity. These predictions can be applied to identify high-risk patients with overweight in a timely manner and aggressive interventions can be implemented to prevent further obesity development.

## Discussion

This study aimed to identify a minimal set of important but most common factors for obesity prediction using the selected optimal machine learning algorithm among 5,236 adults with overweight. The study identified four important factors which can better differentiate overweight subgroups who have a propensity to have obesity from the general overweight population. Moreover, CatBoost is superior to the other six machine learning programs and ranks as the best algorithm. Finally, the application of SHAP (24) in machine learning models solved the problem of poor readability and could better interpret the model established by machine learning and apply it to the early detection, monitoring, and intervention of obesity.

CatBoost is a novel machine learning algorithm with two innovations: Ordered Target Statistics and Ordered Boosting (25). Recently, studies have verified that CatBoost has the best prediction results among all algorithms for all metrics except for specificity for data from various datasets, including those for biochemistry, medicine, and others (25). Traditional biostatistical methods to assess models mainly focus on prediction models for specificity, sensitivity, and AUC. These approaches are mathematically simple to perform but have low clinical relevance. DCA was developed to overcome the limitations of traditional biostatistical approaches and can help assess whether using a model to aid clinical decision-making would improve patient outcomes (26). Thus, DCA was applied to help analyze whether the various models could improve predictive outcomes for patients in this study, especially for the CatBoost model.

In principle, artificial intelligence methods based on machine learning, such as CatBoost, bend to black box models. Compared with traditional models, these black box models exhibit significant advantages in acquiring accurate predictions (27). However, the machine learning models currently in use are not explainable (27). Thus, the SHAP model was developed. In the SHAP model, the SHAP value is a uniform measure of the importance of the features used in machine learning models (16, 28, 29). By attributing output values to the SHAP value of each feature, researchers have performed interpretable analyses of machine learning models (30). Furthermore, the SHAP force plot illustrates the prediction model at an individual level. Through the SHAP analysis of individual samples, we can screen high-risk samples and identify high-risk patients. The results of the interpretability analysis demonstrated the excellent applicability and generalizability of the findings obtained using the CatBoost model.

Based on the CatBoost algorithm, four features were screened, including WC, HC, female sex, and SBP. WC and HC contributed the most to the model. Taking full account of both WC and HC separately, rather than as a ratio measure, has been suggested as a stronger risk predictor of premature death in individuals who are overweight and obese (31). Cameron et al. (32) and Seidell et al. (31) demonstrates that the waist-to-hip ratio (WHR) is a weak predictor of the specific influences of each measurement, as individuals with an identical WHR can have different waist and hip circumferences.

The correlation between WC and all-cause mortality remains a hot controversial topic. As BMI does not reflect the distribution of fat, WC provided further information about the risk of cardiovascular disease, hypertension, and type 2 diabetes, which increases with increasing BMI and WC (33). A prospective observational study conducted by Frank B. Hu suggested a potential causal association between higher WC and all-cause and cardiovascular mortality (34). In accordance with Frank B. Hu, Cerhan et al. also showed that a higher WC is positively associated with higher mortality (35). Hence, they suggested that WC should be evaluated in combination with BMI, even for those in the normal and overweight BMI range in adults, as part of the risk evaluation for obesity-related premature mortality (34, 35).

Moreover, there is increasing evidence that changes in WC might be associated with obesity and additional health outcomes compared to static weight status (34, 35). Cabrera et al. showed that WC was a better indicator of fat mass in older individuals than BMI (36). Aging is usually associated with a higher WC, and redistribution of fat to the abdominal region is often reported in older individuals (37). Therefore, it is important to evaluate long-term trends in WC among adults with overweight.

Waist and hip circumferences measure different aspects of body composition and fat distribution, and have independent effects on CVD factors (32). Cameron et al. showed a strong confounding effect of HC on the correlation between obesity and both cardiovascular and all-cause mortality (32). Moreover, some studies support the powerful association between HC and either heart disease or metabolic disorders, which only becomes apparent after adjustment for WC (38). Narrow hips may reflect less subcutaneous fat, which could have a beneficial influence on the risk factors. However, according to the SHAP plot value in this study, a higher HC reflected a higher risk of obesity among individuals with overweight. There are two potential explanations for this observation. First, narrow hip circumferences may alternatively reflect gluteal atrophy. Second, the average age of the participants included in this study was 55 years, which implied that most participants were middle-aged. Up to 30% of community-dwelling participants older than 50 years live with sarcopenia, an age-related decline in muscle mass (39). Data from the National Health and Nutrition Examination Survey (NHANES) show that 33.5% of females and 12.6% of males over 60 years of age were sarcopenic obese (40). With aging, not only does body fat increase, but it is also distributed differently.

Sex and SBP also were contributors. Our results show that the female sex is a predictive factor for obesity among individuals with overweight. Fat mass, fat distribution, and muscle mass differed between females and males. Males commonly have lower fat mass than females, but have higher insulin resistance due to the abundant fat distributed in the abdominal region (41). Conversely, females commonly have lower body mass-adjusted muscle mass than males (41) and females with type 2 diabetes are at heightened risk for obesity (42). Additionally, SBP is another predictor. Overweight manifests early as autonomic dysregulation (43). Taffe et al. found that an increase in SBP is positively associated with an increase in BMI (44). This is in line with the expectation that SBP, a measure of blood force during ventricular contraction, is influenced more by sympathetic cardiac activity (44). Furthermore, some studies have suggested that the basic cause of high SBP in overweight participants is mainly due to a combination of factors that promote atherosclerosis and systemic vascular resistance (45).

This study has a few limitations. First, this was a single-center, cross-sectional investigation. Future multicenter longitudinal cohort studies should be conducted to verify the accuracy of the model. Second, this study aimed to identify the most common and easily acquired features for predicting obesity. Thus, although diet habits, physical activity, and economic status may be affected, they have not yet been analyzed because of the variety and complexity of these data.

## Conclusion

We described an application based on machine learning and SHAP for an obesity risk prediction model in overweight adults. CatBoost, which was selected as the optimal algorithm, may surpass previous machine learning programs. Furthermore, a combination of interpretative SHAP values and machine learning may be a good approach for identifying disease risk factors for prevention and control. In this study, WC, HC, female sex, and SBP were the top four significant features that could better predict obesity in the overweight population. Consequently, early and efficient preventive measures and treatment strategies, such as lifestyle intervention or pharmacotherapy, should be considered for overweight adults who are predicted to have a higher risk of obesity.

## Data availability statement

The original contributions presented in the study are included in the article/**Supplementary Material**. Further inquiries can be directed to the corresponding authors.

## Ethics statement

The studies involving humans were approved by The Ethics Committee of Fujian Provincial Hospital (Approval No. K2009-12-020). The studies were conducted in accordance with the local legislation and institutional requirements. The participants provided their written informed consent to participate in this study.

## Author contributions

WL: Conceptualization, Data curation, Formal Analysis, Funding acquisition, Investigation, Resources, Visualization, Writing – original draft, Writing – review & editing, Methodology, Project administration. SS: Conceptualization, Methodology, Software, Writing – original draft, Writing – review & editing, Formal Analysis. HH: Visualization, Writing – review & editing, Data curation, Resources. JW: Project administration, Resources, Supervision, Writing – review & editing, Conceptualization. GC: Project administration, Resources, Supervision, Writing – review & editing, Conceptualization, Data curation.
