# Supplementary material for: Predicting risk of obesity in overweight adults using interpretable machine learning algorithms
Source: Front Endocrinol (Lausanne). 2023 Nov 17;14:1292167. doi: 10.3389/fendo.2023.1292167 (PMC10693451; doi:10.3389/fendo.2023.1292167)
Supplement: Supplementary file 1 [file Table_1.docx]

**Supplementary Data 4 Basic information of the participants**

|  | | **Total** | | | **Train** | | | **Test** | | |
| --- | --- | --- | --- | --- | --- | --- | --- | --- | --- | --- |
|  | **Overweight**  **(n=4017)** | | **Obesity**  **(n=1219)** | **Overall**  **(n=5236)** | **Overweight**  **(n=2837)** | **Obesity**  **(n=828)** | **Overall**  **(n=3665)** | **Overweight**  **(n=1180)** | **Obesity**  **(n=391)** | **Overall**  **(n=1571)** |
| **Gender** | |  |  |  |  |  |  |  |  |  |
| Male | | 1793 (44.6%) | 486 (39.9%) | 2279 (43.5%) | 1243 (43.8%) | 327 (39.5%) | 1570 (42.8%) | 550 (46.6%) | 159 (40.7%) | 709 (45.1%) |
| Female | | 2224 (55.4%) | 733 (60.1%) | 2957 (56.5%) | 1594 (56.2%) | 501 (60.5%) | 2095 (57.2%) | 630 (53.4%) | 232 (59.3%) | 862 (54.9%) |
| **Age** | |  |  |  |  |  |  |  |  |  |
| Mean (SD) | | 54.6 (9.85) | 55.1 (9.71) | 54.7 (9.82) | 54.6 (9.89) | 55.1 (9.84) | 54.7 (9.88) | 54.5 (9.77) | 55.1 (9.45) | 54.6 (9.69) |
| Median [Min, Max] | | 54.0 [21.0, 89.0] | 54.0 [32.0, 83.0] | 54.0 [21.0, 89.0] | 54.0 [23.0, 86.0] | 54.0 [32.0, 83.0] | 54.0 [23.0, 86.0] | 53.0 [21.0, 89.0] | 54.0 [34.0, 80.0] | 54.0 [21.0, 89.0] |
| Missing | | 0 (0%) | 1 (0.1%) | 1 (0.0%) | / | / | / | / | / | / |
| **Diabetes** | |  |  |  |  |  |  |  |  |  |
| No | | 3662 (91.2%) | 1066 (87.4%) | 4728 (90.3%) | 2578 (90.9%) | 714 (86.2%) | 3292 (89.8%) | 1085 (91.9%) | 352 (90.0%) | 1437 (91.5%) |
| Yes | | 354 (8.8%) | 153 (12.6%) | 507 (9.7%) | 259 (9.1%) | 114 (13.8%) | 373 (10.2%) | 95 (8.1%) | 39 (10.0%) | 134 (8.5%) |
| Missing | | 1 (0.0%) | 0 (0%) | 1 (0.0%) | / | / | / | / | / | / |
| **MI** | |  |  |  |  |  |  |  |  |  |
| No | | 4003 (99.7%) | 1212 (99.4%) | 5215 (99.6%) | 2829 (99.7%) | 824 (99.5%) | 3653 (99.7%) | 1176 (99.7%) | 389 (99.5%) | 1565 (99.6%) |
| Yes | | 12 (0.3%) | 6 (0.5%) | 18 (0.3%) | 8 (0.3%) | 4 (0.5%) | 12 (0.3%) | 4 (0.3%) | 2 (0.5%) | 6 (0.4%) |
| Missing | | 2 (0.0%) | 1 (0.1%) | 3 (0.1%) | / | / | / | / | / | / |
| **Cerebral.infarction** | |  |  |  |  |  |  |  |  |  |
| No | | 3999 (99.6%) | 1210 (99.3%) | 5209 (99.5%) | 2826 (99.6%) | 821 (99.2%) | 3647 (99.5%) | 1175 (99.6%) | 390 (99.7%) | 1565 (99.6%) |
| Yes | | 16 (0.4%) | 8 (0.7%) | 24 (0.5%) | 11 (0.4%) | 7 (0.8%) | 18 (0.5%) | 5 (0.4%) | 1 (0.3%) | 6 (0.4%) |
| Missing | | 2 (0.0%) | 1 (0.1%) | 3 (0.1%) | / | / | / | / | / | / |
| **Coronary.heart.Disease** | |  |  |  |  |  |  |  |  |  |
| No | | 3909 (97.3%) | 1172 (96.1%) | 5081 (97.0%) | 2757 (97.2%) | 797 (96.3%) | 3554 (97.0%) | 1154 (97.8%) | 376 (96.2%) | 1530 (97.4%) |
| Yes | | 106 (2.6%) | 46 (3.8%) | 152 (2.9%) | 80 (2.8%) | 31 (3.7%) | 111 (3.0%) | 26 (2.2%) | 15 (3.8%) | 41 (2.6%) |
| Missing | | 2 (0.0%) | 1 (0.1%) | 3 (0.1%) | / | / | / | / | / | / |
| **Hypertension** | |  |  |  |  |  |  |  |  |  |
| No | | 3059 (76.2%) | 775 (63.6%) | 3834 (73.2%) | 2165 (76.3%) | 538 (65.0%) | 2703 (73.8%) | 898 (76.1%) | 237 (60.6%) | 1135 (72.2%) |
| Yes | | 954 (23.7%) | 444 (36.4%) | 1398 (26.7%) | 672 (23.7%) | 290 (35.0%) | 962 (26.2%) | 282 (23.9%) | 154 (39.4%) | 436 (27.8%) |
| Missing | | 4 (0.1%) | 0 (0%) | 4 (0.1%) | / | / | / | / | / | / |
| **Peripheral.artery.disease** | |  |  |  |  |  |  |  |  |  |
| No | | 4006 (99.7%) | 1217 (99.8%) | 5223 (99.8%) | 2833 (99.9%) | 828 (100%) | 3661 (99.9%) | 1175 (99.6%) | 390 (99.7%) | 1565 (99.6%) |
| Yes | | 9 (0.2%) | 1 (0.1%) | 10 (0.2%) | 4 (0.1%) | 0 (0%) | 4 (0.1%) | 5 (0.4%) | 1 (0.3%) | 6 (0.4%) |
| Missing | | 2 (0.0%) | 1 (0.1%) | 3 (0.1%) | / | / | / | / | / | / |
| **Hyperlipemia** | |  |  |  |  |  |  |  |  |  |
| No | | 3711 (92.4%) | 1095 (89.8%) | 4806 (91.8%) | 2624 (92.5%) | 750 (90.6%) | 3374 (92.1%) | 1088 (92.2%) | 346 (88.5%) | 1434 (91.3%) |
| Yes | | 305 (7.6%) | 123 (10.1%) | 428 (8.2%) | 213 (7.5%) | 78 (9.4%) | 291 (7.9%) | 92 (7.8%) | 45 (11.5%) | 137 (8.7%) |
| Missing | | 1 (0.0%) | 1 (0.1%) | 2 (0.0%) | / | / | / | / | / | / |
| **Fatty.liver** | |  |  |  |  |  |  |  |  |  |
| No | | 3814 (94.9%) | 1102 (90.4%) | 4916 (93.9%) | 2697 (95.1%) | 743 (89.7%) | 3440 (93.9%) | 1120 (94.9%) | 360 (92.1%) | 1480 (94.2%) |
| Yes | | 200 (5.0%) | 116 (9.5%) | 316 (6.0%) | 140 (4.9%) | 85 (10.3%) | 225 (6.1%) | 60 (5.1%) | 31 (7.9%) | 91 (5.8%) |
| Missing | | 3 (0.1%) | 1 (0.1%) | 4 (0.1%) |  |  |  |  |  |  |
| **Chronic.nephrosis** | |  |  |  |  |  |  |  |  |  |
| No | | 4004 (99.7%) | 1213 (99.5%) | 5217 (99.6%) | 2832 (99.8%) | 822 (99.3%) | 3654 (99.7%) | 1173 (99.4%) | 391 (100%) | 1564 (99.6%) |
| Yes | | 12 (0.3%) | 6 (0.5%) | 18 (0.3%) | 5 (0.2%) | 6 (0.7%) | 11 (0.3%) | 7 (0.6%) | 0 (0%) | 7 (0.4%) |
| Missing | | 1 (0.0%) | 0 (0%) | 1 (0.0%) | / | / | / | / | / | / |
| **COPD** | |  |  |  |  |  |  |  |  |  |
| No | | 3949 (98.3%) | 1198 (98.3%) | 5147 (98.3%) | 2792 (98.4%) | 814 (98.3%) | 3606 (98.4%) | 1158 (98.1%) | 384 (98.2%) | 1542 (98.2%) |
| Yes | | 67 (1.7%) | 21 (1.7%) | 88 (1.7%) | 45 (1.6%) | 14 (1.7%) | 59 (1.6%) | 22 (1.9%) | 7 (1.8%) | 29 (1.8%) |
| Missing | | 1 (0.0%) | 0 (0%) | 1 (0.0%) |  |  |  |  |  |  |
| **Smoking** | |  |  |  |  |  |  |  |  |  |
| No | | 3172 (79.0%) | 966 (79.2%) | 4138 (79.0%) | 2264 (79.8%) | 669 (80.8%) | 2933 (80.0%) | 929 (78.7%) | 304 (77.7%) | 1233 (78.5%) |
| Yes | | 824 (20.5%) | 246 (20.2%) | 1070 (20.4%) | 573 (20.2%) | 159 (19.2%) | 732 (20.0%) | 251 (21.3%) | 87 (22.3%) | 338 (21.5%) |
| Missing | | 21 (0.5%) | 7 (0.6%) | 28 (0.5%) | / | / | / | / | / | / |
| **WC** | |  |  |  |  |  |  |  |  |  |
| Mean (SD) | | 84.8 (5.99) | 93.2 (7.17) | 86.7 (7.22) | 84.8 (5.99) | 93.2 (6.89) | 86.7 (7.13) | 84.6 (5.97) | 93.1 (7.76) | 86.7 (7.42) |
| Median [Min, Max] | | 85.0 [20.0, 107] | 93.0 [67.0, 150] | 86.0 [20.0, 150] | 85.0 [27.0, 107] | 93.0 [68.0, 128] | 86.0 [27.0, 128] | 84.6 [20.0, 103] | 93.0 [67.0, 150] | 86.0 [20.0, 150] |
| Missing | | 1 (0.0%) | 1 (0.1%) | 2 (0.0%) | / | / | / | / | / | / |
| **HC** | |  |  |  |  |  |  |  |  |  |
| Mean (SD) | | 94.6 (4.82) | 101 (5.98) | 96.0 (5.77) | 94.6 (4.92) | 101 (5.65) | 96.0 (5.73) | 94.6 (4.57) | 101 (6.63) | 96.2 (5.85) |
| Median [Min, Max] | | 95.0 [31.0, 154] | 101 [54.1, 140] | 96.0 [31.0, 154] | 95.0 [31.0, 125] | 101 [60.3, 125] | 96.0 [31.0, 125] | 95.0 [79.0, 154] | 101 [54.1, 140] | 96.0 [54.1, 154] |
| Missing | | 1 (0.0%) | 1 (0.1%) | 2 (0.0%) | / | / | / | / | / | / |
| **Osteoporosis** | |  |  |  |  |  |  |  |  |  |
| No | | 896 (22.3%) | 272 (22.3%) | 1168 (22.3%) | 866 (30.5%) | 270 (32.6%) | 1136 (31.0%) | 384 (32.5%) | 121 (30.9%) | 505 (32.1%) |
| Yes | | 2767 (68.9%) | 828 (67.9%) | 3595 (68.7%) | 1971 (69.5%) | 558 (67.4%) | 2529 (69.0%) | 796 (67.5%) | 270 (69.1%) | 1066 (67.9%) |
| Missing | | 354 (8.8%) | 119 (9.8%) | 473 (9.0%) | / | / | / | / | / | / |
| **GFR** | |  |  |  |  |  |  |  |  |  |
| Mean (SD) | | 93.2 (14.3) | 92.3 (14.4) | 93.0 (14.3) | 92.9 (14.4) | 92.1 (14.4) | 92.7 (14.4) | 94.0 (13.8) | 92.8 (14.5) | 93.7 (14.0) |
| Median [Min, Max] | | 95.2 [9.73, 145] | 94.7 [26.7, 149] | 95.2 [9.73, 149] | 95.0 [9.73, 129] | 94.8 [26.7, 122] | 95.0 [9.73, 129] | 95.8 [31.0, 145] | 94.0 [27.0, 149] | 95.5 [27.0, 149] |
| Missing | | 7 (0.2%) | 3 (0.2%) | 10 (0.2%) | / | / | / | / | / | / |
| **HOMA.IR** | |  |  |  |  |  |  |  |  |  |
| Mean (SD) | | 2.14 (2.93) | 2.95 (2.12) | 2.33 (2.78) | 2.17 (3.25) | 2.97 (2.20) | 2.35 (3.06) | 2.08 (1.92) | 2.90 (1.94) | 2.28 (1.95) |
| Median [Min, Max] | | 1.74 [0, 90.6] | 2.49 [0, 33.0] | 1.89 [0, 90.6] | 1.75 [0, 90.6] | 2.54 [0, 33.0] | 1.88 [0, 90.6] | 1.73 [0, 43.4] | 2.40 [0, 19.7] | 1.89 [0, 43.4] |
| Missing | | 8 (0.2%) | 0 (0%) | 8 (0.2%) | / | / | / | / | / | / |
| **FBG** | |  |  |  |  |  |  |  |  |  |
| Mean (SD) | | 5.84 (1.52) | 6.09 (1.61) | 5.90 (1.54) | 5.86 (1.55) | 6.09 (1.67) | 5.91 (1.58) | 5.80 (1.40) | 6.07 (1.44) | 5.86 (1.42) |
| Median [Min, Max] | | 5.53 [3.28, 22.3] | 5.74 [3.36, 17.4] | 5.56 [3.28, 22.3] | 5.54 [3.28, 22.3] | 5.74 [3.43, 17.4] | 5.57 [3.28, 22.3] | 5.52 [3.51, 16.7] | 5.74 [3.36, 17.0] | 5.55 [3.36, 17.0] |
| Missing | | 56 (1.4%) | 24 (2.0%) | 80 (1.5%) | / | / | / | / | / | / |
| **PBG** | |  |  |  |  |  |  |  |  |  |
| Mean (SD) | | 8.14 (3.80) | 8.81 (3.86) | 8.29 (3.82) | 8.17 (3.85) | 8.85 (3.90) | 8.33 (3.87) | 8.03 (3.58) | 8.70 (3.68) | 8.20 (3.62) |
| Median [Min, Max] | | 7.11 [2.17, 34.4] | 7.76 [2.59, 30.1] | 7.21 [2.17, 34.4] | 7.12 [2.17, 34.4] | 7.81 [2.59, 30.1] | 7.22 [2.17, 34.4] | 7.06 [2.27, 28.8] | 7.75 [3.05, 24.0] | 7.21 [2.27, 28.8] |
| Missing | | 65 (1.6%) | 25 (2.1%) | 90 (1.7%) | / | / | / | / | / | / |
| **HbAlc** | |  |  |  |  |  |  |  |  |  |
| Mean (SD) | | 6.01 (1.06) | 6.24 (1.09) | 6.07 (1.07) | 6.03 (1.10) | 6.24 (1.12) | 6.08 (1.11) | 5.97 (0.940) | 6.22 (1.02) | 6.04 (0.967) |
| Median [Min, Max] | | 5.80 [3.00, 14.1] | 6.00 [3.60, 14.0] | 5.80 [3.00, 14.1] | 5.80 [3.00, 14.1] | 6.00 [3.60, 14.0] | 5.80 [3.00, 14.1] | 5.80 [3.40, 14.1] | 6.00 [4.60, 13.4] | 5.80 [3.40, 14.1] |
| Missing | | 4 (0.1%) | 0 (0%) | 4 (0.1%) | / | / | / | / | / | / |
| **HDL** | |  |  |  |  |  |  |  |  |  |
| Mean (SD) | | 1.30 (0.314) | 1.26 (0.284) | 1.29 (0.308) | 1.30 (0.316) | 1.27 (0.290) | 1.29 (0.311) | 1.29 (0.309) | 1.25 (0.268) | 1.28 (0.300) |
| Median [Min, Max] | | 1.28 [0.350, 2.62] | 1.23 [0.370, 2.56] | 1.27 [0.350, 2.62] | 1.28 [0.350, 2.62] | 1.23 [0.370, 2.56] | 1.27 [0.350, 2.62] | 1.26 [0.380, 2.57] | 1.24 [0.560, 2.13] | 1.26 [0.380, 2.57] |
| Missing | | 3 (0.1%) | 2 (0.2%) | 5 (0.1%) | / | / | / | / | / | / |
| **LDL** | |  |  |  |  |  |  |  |  |  |
| Mean (SD) | | 3.07 (0.858) | 3.18 (0.822) | 3.09 (0.851) | 3.08 (0.878) | 3.18 (0.815) | 3.10 (0.865) | 3.03 (0.805) | 3.18 (0.834) | 3.07 (0.814) |
| Median [Min, Max] | | 3.02 [0.440, 10.3] | 3.14 [0.990, 6.69] | 3.05 [0.440, 10.3] | 3.03 [0.660, 10.3] | 3.13 [0.990, 6.25] | 3.06 [0.660, 10.3] | 2.99 [0.440, 7.28] | 3.17 [1.18, 6.69] | 3.04 [0.440, 7.28] |
| Missing | | 5 (0.1%) | 3 (0.2%) | 8 (0.2%) | / | / | / | / | / | / |
| **TG** | |  |  |  |  |  |  |  |  |  |
| Mean (SD) | | 1.86 (1.36) | 2.07 (1.48) | 1.91 (1.39) | 1.85 (1.36) | 2.04 (1.55) | 1.89 (1.40) | 1.88 (1.35) | 2.13 (1.32) | 1.94 (1.34) |
| Median [Min, Max] | | 1.49 [0.300, 15.9] | 1.68 [0.450, 15.7] | 1.53 [0.300, 15.9] | 1.49 [0.310, 15.9] | 1.65 [0.450, 15.7] | 1.52 [0.310, 15.9] | 1.49 [0.300, 11.8] | 1.81 [0.540, 10.7] | 1.54 [0.300, 11.8] |
| Missing | | 12 (0.3%) | 4 (0.3%) | 16 (0.3%) | / | / | / | / | / | / |
| **SBP** | |  |  |  |  |  |  |  |  |  |
| Mean (SD) | | 138 (19.2) | 142 (19.3) | 139 (19.3) | 137 (19.2) | 142 (19.2) | 138 (19.3) | 138 (19.2) | 142 (19.5) | 139 (19.4) |
| Median [Min, Max] | | 136 [62.0, 220] | 141 [0, 228] | 137 [0, 228] | 135 [62.0, 220] | 141 [98.0, 228] | 137 [62.0, 228] | 136 [96.0, 208] | 141 [0, 206] | 137 [0, 208] |
| Missing | | 3 (0.1%) | 0 (0%) | 3 (0.1%) | / | / | / | / | / | / |
| **DBP** | |  |  |  |  |  |  |  |  |  |
| Mean (SD) | | 79.4 (10.5) | 81.7 (11.4) | 80.0 (10.7) | 79.3 (10.5) | 81.8 (11.0) | 79.8 (10.7) | 79.8 (10.4) | 81.7 (12.1) | 80.3 (10.9) |
| Median [Min, Max] | | 79.0 [44.0, 130] | 81.0 [0, 125] | 79.0 [0, 130] | 79.0 [44.0, 130] | 81.0 [49.0, 125] | 79.0 [44.0, 130] | 79.0 [50.0, 122] | 82.0 [0, 121] | 80.0 [0, 122] |
| Missing | | 3 (0.1%) | 0 (0%) | 3 (0.1%) | / | / | / | / | / | / |
| **HR** | |  |  |  |  |  |  |  |  |  |
| Mean (SD) | | 78.5 (11.3) | 80.2 (11.9) | 78.9 (11.5) | 78.4 (11.3) | 80.2 (12.0) | 78.8 (11.5) | 78.9 (11.3) | 80.4 (11.5) | 79.3 (11.4) |
| Median [Min, Max] | | 78.0 [42.0, 142] | 79.0 [50.0, 117] | 78.0 [42.0, 142] | 78.0 [42.0, 142] | 79.0 [50.0, 117] | 78.0 [42.0, 142] | 78.0 [43.0, 126] | 81.0 [54.0, 116] | 78.0 [43.0, 126] |
| Missing | | 3 (0.1%) | 1 (0.1%) | 4 (0.1%) | / | / | / | / | / | / |

WC: waist circumference; HC: hip circumference; GFR: glomerular filtration rate; FINS: fasting insulins; HOMA IR: homeostasis model assessment of insulin resistance; FBG: fasting blood glucose; PBG: 2-h postprandial blood glucose; HbAlc: glycosylated hemoglobin; HDL: high-density lipoprotein cholesterol; LDL: low-density lipoprotein cholesterol; TG: total triglyceride; SBP: systolic blood pressure; DBP: diastolic blood pressure; HR: heart rate
