# Supplementary figures and images for: Predicting risk of obesity in overweight adults using interpretable machine learning algorithms
Source: Front Endocrinol (Lausanne). 2023 Nov 17;14:1292167. doi: 10.3389/fendo.2023.1292167 (PMC10693451; doi:10.3389/fendo.2023.1292167)

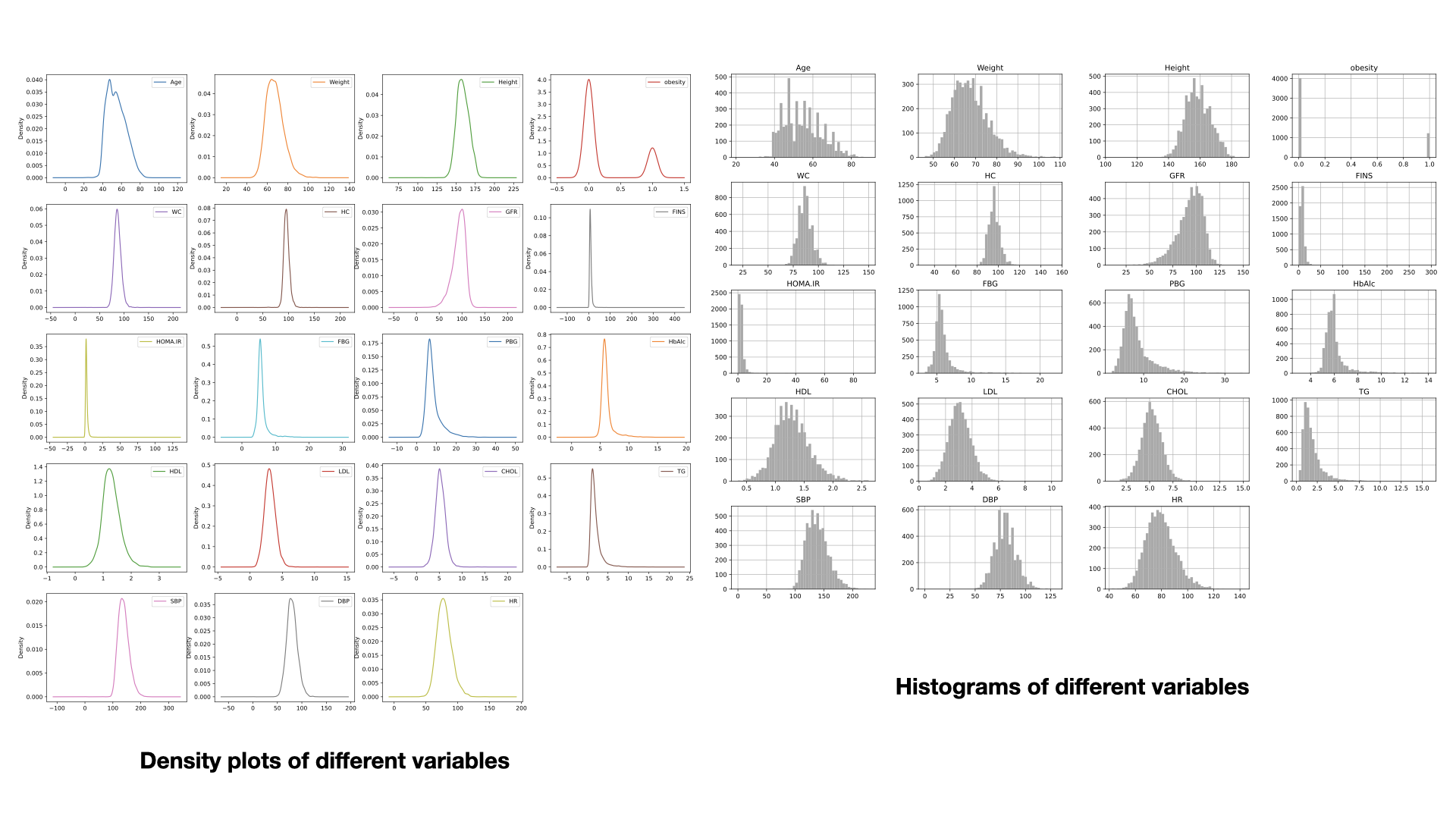

Supplement: Supplementary file 2 [file Image_1.tiff]

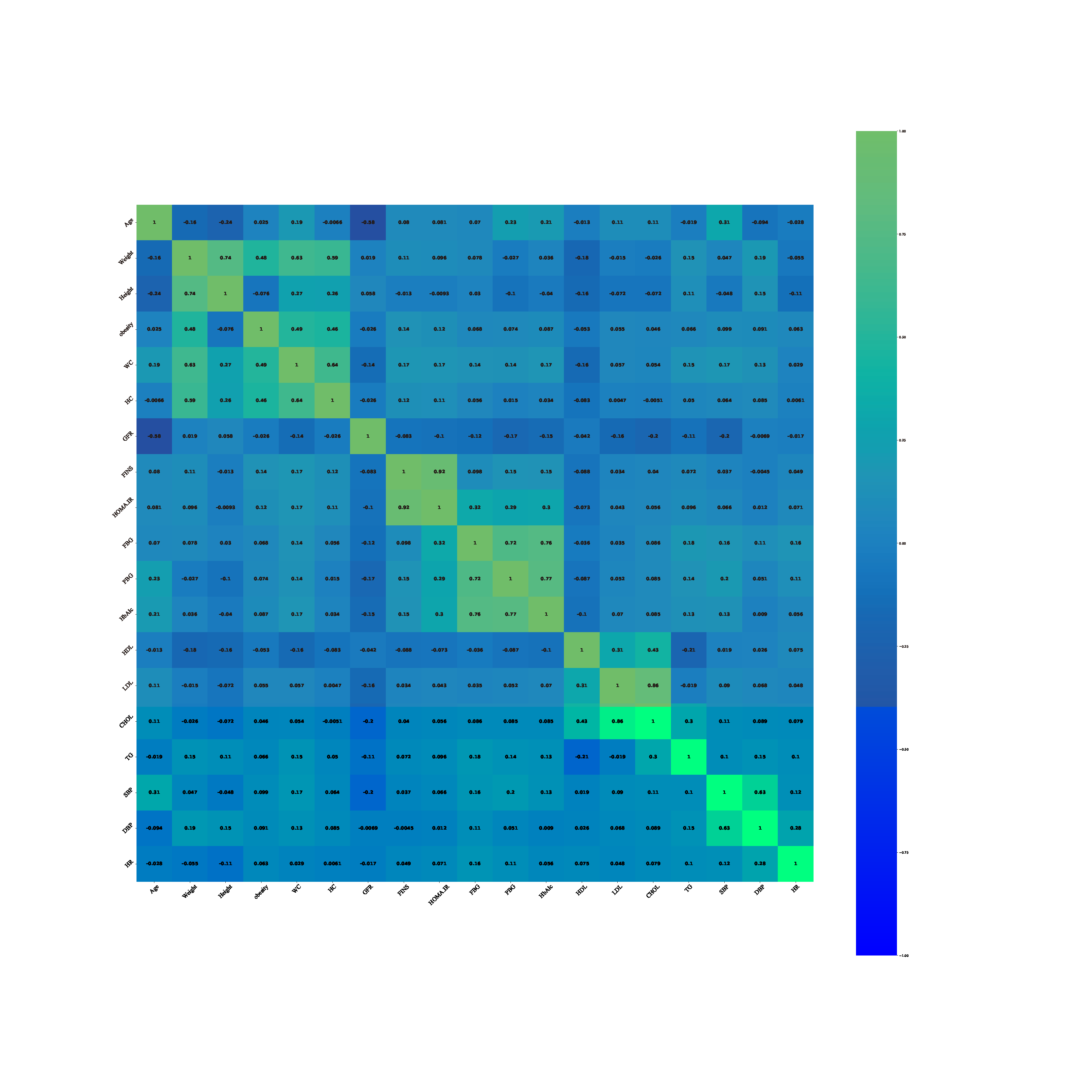

Supplement: Supplementary file 3 [file Image_2.tiff]

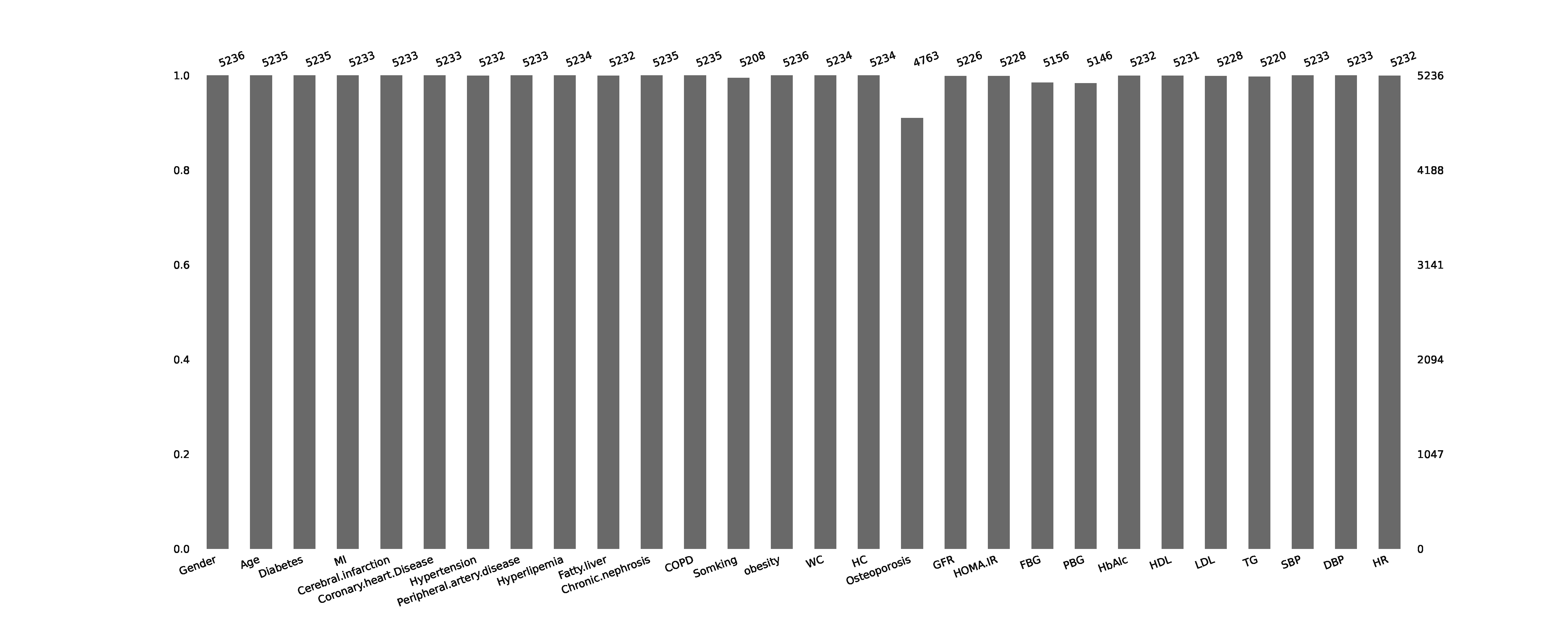

Supplement: Supplementary file 4 [file Image_3.tiff]
